# Digital solidarity and adaptation: applications developed for refugees in host countries

**DOI:** 10.3389/fsoc.2024.1479288

**Published:** 2024-10-31

**Authors:** Özlem Özdemir, Melih Görgün, Elif Başak Sarıoğlu

**Affiliations:** ^1^Faculty of Communication, Istanbul Aydin University, İstanbul, Türkiye; ^2^Department of Political Science and International Relations, Yeditepe University, İstanbul, Türkiye; ^3^Communication Faculty, Marmara University, İstanbul, Türkiye

**Keywords:** refugee, application, international migration, adaptation, digital solidarity, locative media

## Abstract

The scale of international migration is growing day by day, and the issue of refugees constitutes a significant agenda item for all countries. In today's digital age, characterized by technological advancements, refugees are also benefiting from the advantages of technology. With various applications downloaded to their mobile phones, refugees can directly access all kinds of information about the countries they are heading to and even communicate with each other to share experiences. This study explores how mobile phone applications can be developed to serve the refugees in the familiarizing themselves with the receiving culture. In the study, a total of 10 applications prepared for refugees on Android and iOS platforms, selected by random sampling and purposeful sampling methods, were examined. These applications were analyzed using the qualitative content analysis method. As a result, when the applications developed in host countries were examined, it was observed that the most needed basic information for refugees, such as housing, education, finding a job, health, language, daily words, legal documents, rules, and fundamental rights, was provided. Additionally, in the 10 refugee applications examined, Global Positioning System (GPS) and Geographic Information System (GIS) were used as locative media for mapping locations. Location-based data and maps that show where refugees are located are extremely important for them. These maps are believed to help reduce stress, make them feel safe, and facilitate the adaptation process by showing refugees the nearest shelters, dining places, hospitals, transportation, and asylum locations.

## 1 Introduction

Throughout history, people have migrated to unfamiliar regions where they feel safer due to various reasons such as war, conflict, hunger, drought, climate change, unemployment, violence, ethnic cleansing, and genocide. Consequently, migration studies represent a field that brings together different disciplines such as security, communication, anthropology, sociology, international relations, political science, and economics.

The discipline of human mobility studies has a long-standing history. Classical migration theories suggest that the decision to move from one place to another depends on the push and pull factors in the individual's home country and the destination country. Push factors, such as conflict, war, hunger, poverty, inequality, and unemployment, which are the major reasons for international migration, force people dreaming of a better future for themselves and their families to leave their countries (UN, 2016, p. 2).

On the other hand, pull factors, such as improved quality of life, security, a promising future, job opportunities, education, and healthcare, influence individuals to settle in another country. Therefore, human mobility carries many positive and negative connotations. Positively, it is defined as progress, freedom, opportunity, and modernity, while negatively, it is associated with threats, insecurity, deviance, and exploitation (Cresswell, 2006, p. 1–2, 27–28). Migration, as a form of mobility that encompasses both aspects, is one of the significant realities of today. Hence, the phenomenon of global migration has become a situation that touches almost every corner of the world and often creates distinctions between emigrating countries, transit countries, and host countries.

Although international migration has occupied a significant place on the world agenda in the last two decades, its prominence in humanitarian discussions stems from the events in Syria. Since the onset of the Syrian civil war in 2011, 11 million Syrians have been forced to leave their homes; millions fleeing conflict have migrated to neighboring countries or Europe. Europe, which has witnessed the largest migration since World War II, received a total of 1 million refugees. In Germany alone, 300,000 people applied for asylum, while Sweden received 100,000 applications. The number of Syrians who arrived in Türkiye is 3,606,208 (UNHCR, 2019; Syrian Refugees, 2016). Moreover, according to official figures from the United Nations High Commissioner for Refugees (UNHCR, 2019), 68.5 million people worldwide have been displaced. This figure includes 40 million internally displaced persons, 25.4 million refugees, and 3.1 million asylum seekers. Globally, one in every 122 people is either displaced, a refugee, or an asylum seeker. 68% of refugees come from Syria, Afghanistan, South Sudan, Myanmar, and Somalia. The countries hosting the most refugees are Iran, Türkiye, Uganda, Pakistan, and Lebanon.

Forced migration due to war, conflict, natural disasters, human rights violations, famine, hunger, or violence is among the reasons for becoming a refugee. Refugees subjected to forced migration face several dangers during irregular migration. The first of these dangers is the risk of losing their lives during arduous land or sea journeys. The second is the exposure to violence, and the third is the danger of being defrauded by smugglers. The concern brought by danger and anxiety motivates refugees to gain more information about their destination (Gadarian and Albertson, 2014).

With the advancement of digital technologies, migration studies have gained a new dimension. The current conditions, referred to as the digital age, not only affect migration but also transform it. Today, people have the ability to interact digitally regardless of their location. This situation, which ensures digital integration, is also applicable to refugees. Refugees, whether forced or voluntary, can now obtain information about the countries they wish to go to or dream of, even before arriving there, thanks to digital communication technologies. In this respect, smartphones, which have become a kind of life kit for a refugee, are particularly important. Through applications downloaded to their mobile phones, refugees can easily reach the routes they want to go, gain information about countries, communicate with each other, learn the necessary information, and facilitate their adaptation to the country. Location-based applications developed for refugees, GPS maps, GPS-integrated devices, applications, and social media significantly impact refugees' decisions to migrate to other countries, choose migration routes, or apply for asylum (Alencar et al., 2018, p. 9–12). Techniques we used to estimate our direction and location, such as maps, stars, or compasses, have been replaced by GPS with the advancement of digital technologies. GPS is a space-based navigation system that provides location and time information to users under all weather conditions, anywhere in the world, with an unobstructed line of sight to four or more GPS satellites. With a wide range of applications, GPS is also used for navigation in airplanes, ships, cars, and trucks. Moreover, the system provides continuous real-time, 3D positioning, navigation, and timing worldwide. GPS answers a total of five questions related to location, navigation, and time. These are: Where am I? Where am I going? Where are you? How can I get there most easily? When can I reach there?

In this context, some applications developed for refugees were examined. The research highlights the importance of mobile applications in terms of solidarity and integration. At this point, the significance of the research becomes evident. It is thought that evaluating the applications from the perspective of refugees will contribute to both digital international migration and communication studies. The research seeks to answer the following research questions:

1. Which are the mobile apps that have been used for refugees' adaptation?
2. Do mobile phone applications developed for refugees in host countries meet the needs of refugees?
3. Do the applications developed for refugees include locative media?

## 2 Literature review

### 2.1 International migration

Migration is as old a phenomenon as human history itself. Throughout the years, people have moved across different geographies, crossing continents and oceans, creating various civilizations, and shaping history. According to the International Organization for Migration (IOM), migration is defined as “*The movement of a person or a group of people across an international border or within a State. It is a population movement, encompassing any kind of movement of people, whatever its length, composition, and causes; it includes migration of refugees, displaced persons, economic migrants, and persons moving for other purposes, including family reunification*” (IOM, 2019).

International migration is a very dynamic, complex, and multifaceted phenomenon that is constantly changing. It takes various forms in different contexts and develops in diverse ways. It is influenced by the conditions of the context in which it occurs and undergoes transformations through interaction with changes in these conditions (IOM, 2020). Therefore, the concept of migration includes a broad scope of discussion and examination that concerns historical, general, contemporary, and future-oriented fields, which are also subjects of philosophy as part of human mobilization and change (Tuncer, 2021).

In most discussions about migration, the starting point is often numerical data. Understanding numerical changes, emerging trends, and changing demographic information related to global, social, and economic transformations, such as migration, helps us understand the changing world we live in and plan for the future (IOM, 2020).

For a long time, international migration was confined to a specific field of study, but after the 1990s, it began to be addressed and evaluated globally by different disciplines. The formation of the nation-state and the definition of borders led to the emergence of the concept of international migration alongside the nation-state. Until the 1990s, international migration could be regulated by national policies. Since many of these barriers were created by nation-states, it is evident that they are products of the Westphalian period, during which the importance of the nation-state was emphasized. It is known that before this period, people could move quite freely across geographical spaces. With the emergence of the nation-state, these movements began to be examined more meticulously and carefully. To limit, control, and prevent the free movement of people across geographical spaces, much stricter restrictions were introduced.

In the 1990s, the dissolution of the Soviet Union after the Cold War led to the emergence of new states in Eastern Europe; ethnic conflicts and other related events in many regions, especially in the Balkans, the Middle East, and Africa, resulted in significant humanitarian crises and intense migration waves. After the 1990s, due to the inadequacy of immigration policies and their failure to solve existing problems, immigration policies began to collapse. Consequently, migrants who could not be regulated within a specific legal framework due to the inadequacy of existing organizational arrangements were referred to as illegal or irregular migrants (Gök, 2016). Post-Cold War security approaches have evolved into a global perspective that includes not only state-centered international security but also individuals, ethnic or religious groups, minorities, international companies, non-governmental organizations, and the international system.

Migration, when considered within security approaches, can generally be evaluated from three different perspectives. Firstly, it is considered a solution to the insecure environments that mobilize masses. Secondly, regardless of the causes, it can be addressed in the context of insecurities encountered during the movement from the source country to the destination country and experienced in both the destination and transit countries. Finally, it involves the attitudes of transit and destination countries that perceive migration and refugee influxes as security threats (Özdemir, 2019). Whether forced or voluntary, migrations offer new opportunities on one hand while causing significant suffering on the other.

Migration continues to shape the world globally today. At the end of 2023, there were 117.3 million forcibly displaced people worldwide. Of these, 68.3 million are internally displaced people, 6.9 million are asylum-seekers, 5.8 million are other people in need of international protection, and 37.6 million are refugees (UN, 2024). Discussions about migration often involve a combination of push and pull factors. Push factors include motivations of migrants, such as unemployment, low wages, and significant upheavals like war, famine, political persecution, or economic collapse in their home countries. Pull factors include favorable immigration policies in the target country, high wages, lower unemployment rates, formal and informal networks available to migrants, the need for labor, and cultural and linguistic similarities between the source country and the target country.

The restriction and control of human mobility, which has increased with irregular migration, have become one of the most important agenda items today. The term irregular migration describes individuals who do not comply with some aspects of immigration law and regulations. Although often referred to as illegal immigrants, many prefer the terms “irregular” or “undocumented” migrants. Irregular migration encompasses various forms of irregularity. For example, individuals who enter a country legally but do not leave when their legal entry period expires; those who enter and work without legal permission or fail to renew their work permits, including their children; and those whose asylum applications are rejected and whose legal stay period has expired, fall under the category of irregular migrants (Migrant Work and Migrants' Rights Network, 2009). This demonstrates the fluidity between concepts and categories while highlighting the difficulty in defining them. Transit migration, in its broadest sense, refers to being at an intermediary stop on the migration journey, indicating that the target destination has not yet been reached. It signifies a point passed en route from the sending country to the intended destination country. In the past, this situation was described as step-by-step migration within an approach that viewed migration as a process developing in stages. Although there have been definitional differences over time, throughout history, people have migrated to unfamiliar geographies where they feel safer for various reasons.

### 2.2 Irregular migration and refugees

Research conducted by academics, non-governmental organizations, and governments on irregular migration reveals the diverse ways in which people's migration becomes irregular. Therefore, human mobility studies have a long history. International migration is a very dynamic, complex, and multifaceted field of research. Push factors, such as conflict, war, hunger, poverty, inequality, and unemployment, which are among the major reasons for international migration, force people dreaming of a better future for themselves and their families to leave their countries (UN, 2016). On the other hand, pull factors, such as improved quality of life, security, a promising future, job opportunities, education, and healthcare, also encourage people to settle in another country. Human mobility and consequent migration have led to the emergence of many refugees, asylum-seekers, and migrants. The phenomenon of global migration has become a reality that touches almost every corner of the world and often creates distinctions between emigrating countries, transit countries, and host countries. Addressing migration as a field that brings together different disciplines will make management in this area more effective.

Irregular migration represents the portion of cross-border human movements that states cannot control or monitor. Frequently featured in the media, irregular migration is not limited to migrant smuggling but encompasses a range of phenomena, including human trafficking, individuals who legally enter a country and overstay their visas, and the illegal or forced labor of legal or illegal migrants.

Irregular migration concerns sending, receiving, and transit countries, as well as their legal frameworks, law enforcement agencies, and non-governmental organizations. Each geographical region has expanded and redefined concepts related to irregular migration, such as refugee, migrant, or asylum-seeker, according to its conditions and events. It is evident that developing a common language and understanding regarding irregular migration and its concepts on an international scale and conducting joint studies on this issue is quite challenging. According to the International Organization for Migration (IOM, 2019), the term refugee, which is often the first concept that comes to mind when discussing irregular migration, is defined as:

“*A person who qualifies for the protection of the United Nations provided by the High Commissioner for Refugees (UNHCR), in accordance with UNHCR's Statute and, notably, subsequent General Assembly's resolutions clarifying the scope of UNHCR's competency, regardless of whether or not he or she is in a country that is a party to the 1951 Convention or the 1967 Protocol – or a relevant regional refugee instrument – or whether or not he or she has been recognized by his or her host country as a refugee under either of these instruments*.”

At the end of 2023, there were 117.3 million forcibly displaced people worldwide, 37.6 million of whom are refugees (UN, 2024).

In the last 10 years, at least 100 million people have been forced to leave their homes, either within their country or across borders, seeking refuge. Forced displacement has remained high on the international agenda in recent years and continues to make headlines worldwide (Tables 1, 2). Several major events have been the primary causes of mass displacements over the past decade, including the Syrian civil war, the humanitarian crisis in South Sudan, the war in Ukraine, the arrival of refugees and migrants in Europe via sea routes, the massive influx of refugees from Myanmar to Bangladesh, the crisis in the Sahel region of Africa where conflict and climate change endanger many communities, conflicts and security concerns in Afghanistan, Iraq, Libya, and Somalia, internal displacement in Ethiopia, violence in the Democratic Republic of Congo, and the major humanitarian crisis in Yemen (UNHCR, 2020). These global crises will likely lead to more extensive measures being taken regarding irregular migration in the coming years.

**Table 1:** Refugee data finder.

**73% originate from just five countries**	**Million**
Afghanistan	6.4
Syrian Arab Republic	6.4
Venezuela	6.1
Ukraine	6.0
South Sudan	2.3

Source: UN (2024).

**Table 2:** Refugee data finder.

**39% hosted in five countries**	**Million**
Islamic Republic of Iran	3.8
Türkiye	3.3
Colombia	2.9
Germany	2.6
Pakistan	2.0

Source: UN (2024).

### 2.3 Mobile technologies and digital migration

The international media's focus on the drowning deaths of Syrian refugees, especially women and children, attempting to reach Europe illegally via sea routes, has drawn attention from academics, politicians, journalists, and non-governmental organizations to the dangers brought by migration. Those who showed social sensitivity to the situation took action by developing applications to help refugees and accelerate the integration process. Digitalization speeds up the integration process. It is argued that refugees need not only a physical but also a digital infrastructure while heading to safer places (Latonero and Kift, 2018). Social media, smartphones, and similar digital network technologies are expanding to encompass the digital transition infrastructure. These are socio-technical domains where refugees, non-governmental organizations, governments, and companies interact with each other and with new technologies. At the same time, this digital infrastructure is also used for monitoring and controlling refugee mobility. Particularly within the framework of European border policies, digital controls over refugee movement and identity have begun (Latonero and Kift, 2018). Because international migration is rapidly digitalizing with the advancement of technologies, digital technologies directly impact refugee mobility. Refugees learn all kinds of information about the country they will go to through social media and websites before leaving their own countries. In this situation, where international migration has gained a new dimension, refugees have started to benefit from the advantages of technology for a better future. For refugees, a smartphone has become more than just a device to communicate with family or friends; it provides opportunities for planning a future in a foreign country, ensuring travel safety, staying informed, socializing, determining migration routes, and easily accessing information. Therefore, “the smartphone actually provides a big picture for researchers” (Ram, 2015).

In the past few years, smartphones and digital technologies (especially software applications-mobile apps) have inevitably become effective tools for those fleeing war in their countries. Smartphones and online platforms offer solutions to various obstacles faced by refugees, including navigation and GPS, translation, communication, housing, and money management (Table 3). Applications like Google Maps, Facebook, WhatsApp, or Bitcoin have features that can act as intermediaries for refugees seeking a better life outside their home countries (Evans et al., 2017). Additionally, non-profit organizations aiming to help refugees have created new jobs with the help of new technologies. Leading the way is the non-profit company Techfugees. Techfugees, an international technology community, is a non-profit organization that coordinates technological responses to the needs of refugees, asylum seekers, and displaced people. According to the organization, smartphones provide refugees with five benefits: access to rights and accurate information, education, health, work, and social integration (Techfugees, 2019).

**Table 3:** Mobile apps for refugees.

**Name of the app**	**Name of The organization**	**Content area**	**Languages**	**Countries where it's used**	**Mobility, locative media and global positioning system**
Love-Europe	Jesus.net-Foundation, Agape Europe, IAFR, Top Mission, SEA Taskforce Flüchtlinge, Agape international, Groningen Verwelkomt	Language, accommodation, transportation, health, weather	English, Arabic, Farsi, German, Greek, Turkish, Tigrinya, Dutch, French, Tamil, Spanish, Italian	Netherlands, Switzerland, France, Greece, United Kingdom, Austria, Portugal, United Kingdom, Cyprus, Bosnia and Herzegovinaand Malta	Yes
Refugee Buddy	Red Cross	Location of schools, food distribution, health centers, Asylum Seekers and Immigrants Solidarity Associations	English, Arabic, French and Tigrinya	Canada, Cyprus and Norway	Yes
REFAID	LifeSpots	Location of schools, food distribution, health centers, Asylum Seekers and Immigrants Solidarity Associations	English, Arabic, Persian, Turkish	Europe, Türkiye, USA	Yes
Find Hello	USAHello	Employment, health, immigrant and refugee rights, education, scholarships, English classes, finding housing, food distribution sites, refugee associations, citizenship information, child and youth services, location of emergency services	English, Arabic, Spanish	USA	Yes
I Stand With Refugees	Associations, charities and organizations	Translation, legal rights, job opportunities, family reunification, language training, first aid and fundraising	English	England	Yes
Integreat	Daniel Kehne	Health, asylum, employment, education and legal rights	German, English, Arabic, Persian and French	Germany	Yes
OKA	Instituto Igarape	Housing, food resources, education, transportation, legal aid, employment and health services	English, Spanish, Portuguese, French	Brazil	Yes
Merhaba Umut	Turkcell	Alphabet, emergency, meeting, health, transportation, food, professions, accommodation	Turkish, Arabic	Türkiye	Yes
I'MAPPY	Center for Asylum and Migration Research (İGAM)	placement of migrant workers access to social and basic information	Italian, Lithuanian, Greek, Arabic, Turkish, and English	Türkiye	Yes
MIGRANT INFORMATION CENTER (MIHUB)	University of Nicosia, CARDET (Center for Applied Research and Development in Educational Technology), and Cyprus University of Technology	Housing, education, health, rights and responsibilities, work, learning English, learning Greek, travel, social benefits, counseling		Cyprus	Yes

Source: Danish Refugee Council (2016), Igarapé Institute (2020), I'MAPPY (2020), INTEGREAT (2020), Love-Europe (2019), Merhaba Umut (2020), MiHUB (2019); OKA (2020), REFAID (2019), Tarjimly (2019), and Turkcell (2020).

Applications aimed at social integration, beyond physical integration, provide refugees with various conveniences, including location data, health, safety, language, dining, family reunification, finding friends, gathering at specific locations, map information, travel safety, weather, housing, legal processes, and refugee rights. Therefore, staying connected through smartphones and social media is of vital importance for refugees. These applications enable refugees to have foresight about their dream destinations before arriving. The locational data, geographic information, and social media communications provided in the applications influence and encourage those who decide to migrate. In some cases, individual migration actions transform into collective actions. As Appadurai (1990) describes as an “imaginary community,” a particular group dreams of living elsewhere due to collective reading, criticism, and enjoyment facilitated by mass media. In this scenario, collectively imagined mobility becomes a collective action.

### 2.4 Mobility, locative media, and global positioning system

Studies on mobility and locative media, along with the introduction of GPS (Global Positioning System) and GIS (Geographic Information System) into mobile devices, continue to discuss how the social construction of place, space, and location is affected (De Souza e Silva and Frith, 2012; De Souza e Silva and Sheller, 2015). Location-based social networks now track and monitor the movements of individuals and objects by tagging their geographic coordinates through space (Duarte, 2015, p. 65). Duarte emphasizes the need to research locative media applications like GPS and GIS when examining topics such as circulation, human mobility, or migration. According to the author, mobile and location-based applications should be included in academic studies as they facilitate overcoming seemingly insurmountable national borders, enabling communication among those deciding to migrate, and interacting with capital, information, and ideas through mobile devices. With technological advancements, borders should be considered hybrid spaces (Duarte, 2015).

Many refugees digitize the international migration process by leveraging innovations brought by the digital age. Thanks to innovations in information and communication technologies, refugees gain prior knowledge about the countries they wish to migrate to via social media, websites, and applications (Diminescu, 2008; Danish Refugee Council, 2016; Schapendonk and van Moppes, 2007). Millions of computers and mobile devices are interconnected via satellites. This extensive internet network, with location uploads containing data sources, makes physical locations part of the internet network. This creates a comprehensive map of where we are. Thus, both users and those tracking them learn their locations with the help of GPS-compatible devices. Refugees, becoming dependent on digital infrastructure, can instantly access desired data and other refugees' experiences regardless of place and time. Consequently, refugees use technology to plan their futures (Kountze, 2017).

Refugees' dependency on technology has also mobilized large corporations. Yahoo and Google have started investing in refugee-related issues. For instance, Google has developed a website to help refugees access useful information both during migration and in host countries. The site, named Crisis Information Center, is designed to provide critical information to asylum-seeking refugees worldwide during their journeys (Al Jazeera, 2015). The United Nations, recognizing the importance of the internet, social media, GPS, and locative media for refugees, collaborates with the private sector, non-governmental organizations, and refugee-hosting countries to provide conveniences to refugees. It is quite challenging for refugees to access these digital technological developments on their own. Therefore, allowing refugees to benefit from what the United Nations calls the digital revolution is a significant development in terms of humanitarian aid. From this perspective, the opportunities offered by the internet and smartphones in the fields of education, health, security, and communication help refugees feel secure (UNHCR, 2016, p. 8, 17).

Smartphones have particular importance for refugees. Thanks to both general and refugee-specific applications installed on smartphones, refugees can obtain information about the health system, the attitude toward refugees, weather conditions, border security, the attitudes of border security forces, travel expenses, the education system, job opportunities, accommodation, and asylum rights of the country they plan to migrate to before they even migrate. Location-based applications make the invisible visible, affecting personal mobility (Frith, 2013). The information gained by communicating with other refugees or voluntary users of these applications can change the migration process or route. Furthermore, a refugee with a mobile phone and language skills has a different status within the group. The refugee chosen as the leader of the group decides the next route and guides the group (Gillespie, 2016, p. 46).

According to a news report by McLaughlin (2015) in The Irish Times, hundreds of thousands of refugees wishing to reach Western Europe via Türkiye, Greece, Macedonia, Serbia, and Hungary communicate with each other via social media through smartphones to pass the border safely without being seen by border security forces, find smugglers to help them cross the border, arrange accommodation, and learn about the attitudes of governments toward refugees. The report mentions that a 20-year-old Afghan refugee, being the only one in the group with a smartphone, is seen as the group leader. The Afghan youth communicates with other refugees, sending location data to indicate dangerous areas and the presence of security forces, thereby changing the migration route. Having a smartphone elevates the youth to a leadership position, turning him into an opinion leader. In short, having a smartphone reduces uncertainty by providing information about the place. The author also mentions, based on information from a volunteer in Hungary, that before reaching any country, refugees inform each other via social media about where to stay, where to eat, and which areas are safe.

For refugees, the use of GPS and social media has both positive and negative aspects. The positive aspects include maintaining communication between family and friends who remain in their home countries or have previously migrated to developed countries, being informed about safe migration routes, sending location data in case of danger, and gaining knowledge about the destination country (Dekker et al., 2018). For example, a map developed for refugees in Mexico under the title of a safe migration map shows high-crime and dangerous areas, directing refugees to safer routes (Raftree, 2014). A news report on the NPR (2015) website titled “Europe's arduous journey, supported by applications and the internet” discusses how Middle Eastern refugees used Viber, WhatsApp, Facebook Messenger, Skype, GPS, and Google Maps to reroute to Croatia and Serbia after Hungary closed its border gates and increased security measures.

According to the joint views of refugees, United Nations officials, and non-governmental organization workers, digital connectivity is the most effective tool for refugees to report any danger, request assistance, determine routes, and access information (food distribution, health services, etc.). Therefore, the smartphone serves as a kind of protection tool (UNHCR, 2015, 2016, p. 12).

In summary, smartphones, applications installed on these phones, map applications, and locative media are vital for ensuring the safety of refugees on difficult and dangerous migration routes. As previously mentioned, applications developed by the United Nations, governments, and volunteers help refugees feel secure but can also be the subject of discussions. According to De Souza e Silva and Frith (2010, p. 503–510), location awareness can be negative or positive depending on its purpose of use. It is considered positive if used for tracking and monitoring for child safety, but negative if used by governments or companies for surveillance and monitoring. So, are the applications developed to control refugees or to ensure their safety? This question was brought to the agenda at a meeting held in January 2016 on the island of Lesbos in Greece. EU countries began expressing their concerns about their inability to control the large number of refugees arriving in Europe via dangerous routes. The main reason is that millions of refugees trying to reach Europe die or disappear during migration. Therefore, the European Union Border Protection Agency (Frontex) asked information technology companies to design smartphone applications and databases to monitor and control refugees arriving in Europe. The primary reasons behind this request are to control refugees' access to food and accommodation via a smart card identity system and to provide useful information to refugees about sea crossings and conditions through applications downloaded to their phones. At the same meeting led by Frontex and attended by major information technology companies such as Securiport LLC, Crossmatch, Unisys, Thales, and 3M, discussions were also held on how to control refugee migration with the help of technology. Unisys officials mentioned that their technology could help prevent some migrants from coming to Europe, preventing other refugees from making dangerous sea journeys and reducing the role of smugglers. They also stated that their plan would start monitoring as many refugees as possible before they leave conflict zones or while they are in refugee camps near these zones (Taylor and Graham-Harrison, 2016).

The topics discussed at the meeting caused reactions from refugee support groups and some non-governmental organizations. Companies would be able to guide, monitor, and track refugees through applications loaded on smartphones in refugee camps or gathering places. According to Carrera (2007), this situation aims to create a common migration policy to manage illegal mobility as part of Europe's border security policy, especially through location-based technologies to track and manage individuals. For this purpose, technological developments in bio-informatics security and remote surveillance form the infrastructure for controlling mobility at borders. However, it should be noted that the more high-tech governments use for border security, the more refugees use technology to cross borders (Vukov and Sheller, 2013).

## 3 Applications developed for refugees

In the study, a total of 10 applications prepared for refugees on Android and iOS platforms were used by employing both purposive sampling and random sampling methods to meet the requirements of the research. Purposive sampling is a very useful method for researchers who want to answer a specific goal or research question. This sampling method is used to reach richer data and increase the credibility of the research (Flick, 2014). The applications selected by sampling in the study were analyzed using qualitative content analysis. Qualitative content analysis is one of the most commonly used research methods in communication studies. Therefore, it can be applied to all types of communication content. Qualitative content analysis is a research method in which the content of a communication tool is systematically researched, interpreted, and analyzed. With this method, information, topics, and symbols in written documents or other communication environments are analyzed and interpreted. This can also be defined as an effort to make some inferences about the interpreted content. The identification, interpretation, summarization, and detection of the messages contained in the obtained findings is a technique of great importance. Through the findings, views and suggestions are put forward by reaching the context (Neuman, 2018).

## 4 Findings

### 4.1 Love-Europe

**Figure 1:**
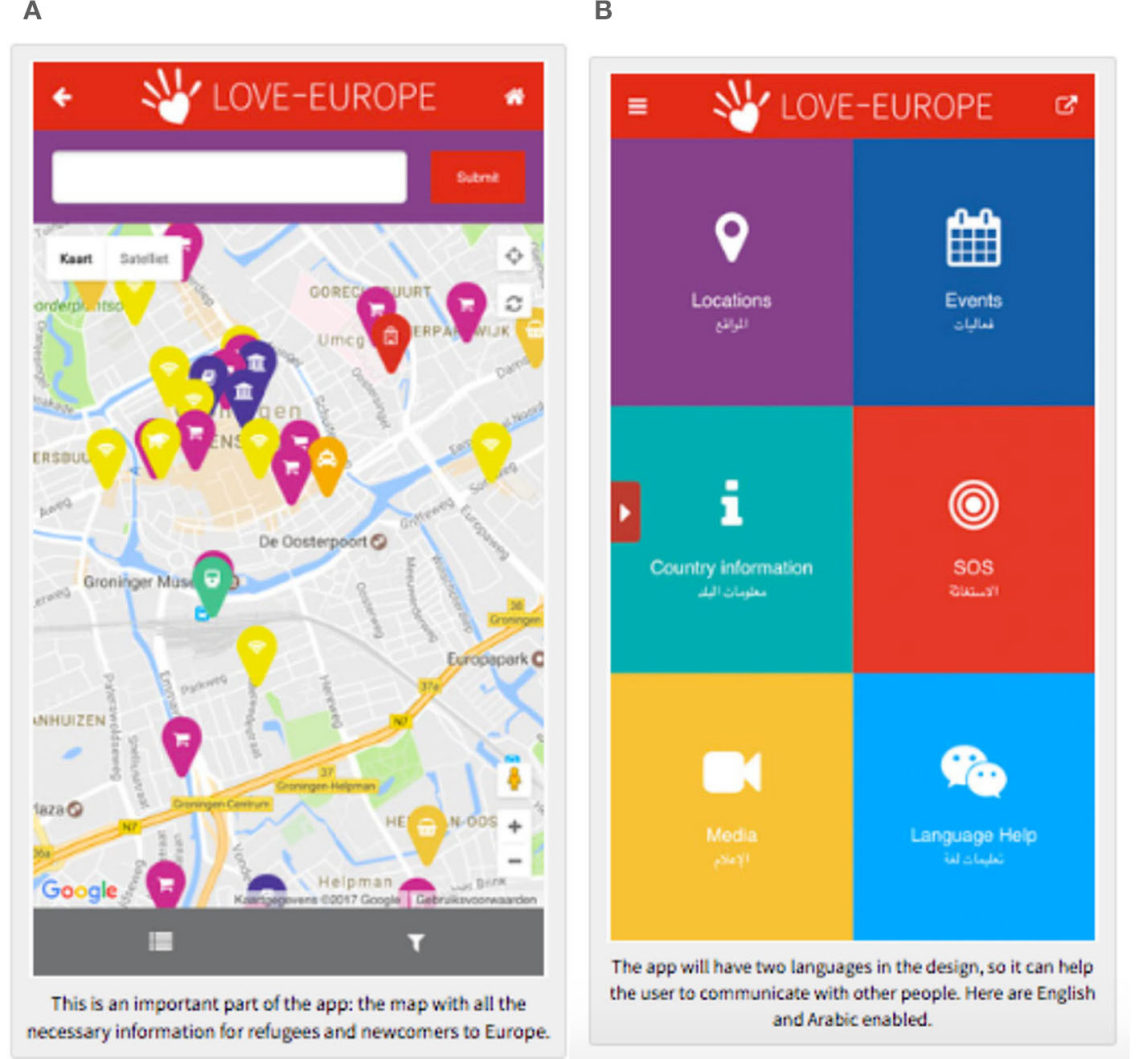
Love Europe.

Within the scope of the study, the Love-Europe application (Figures 1A, B) was examined. It is a project prepared through the collaboration of various organizations (Jesus.net-Foundation, Agape Europe, IAFR, Top Mission, SEA Taskforce Flüchtlinge, Agape International, Groningen Verwelkomt) in European countries. The Love-Europe (2019) application logo is depicted as a helping hand in the shape of a heart. The heart-shaped helping hand logo expresses their willingness to welcome refugees warmly and extend a helping hand from the heart, showing care for them. The Love-Europe application, which also has a website, describes its founding purpose as follows:

“Since 2015, thousands of refugees have been flocking to Europe every day. Many people are fleeing their own countries due to war, risks, and hunger. We see many people coming from the Middle East, Africa, and East Asia in Europe. Although they love the lands where they grew up, it is not possible for many to return safely to their homeland. Since most refugees arrive in Europe with a mobile phone, we want to offer them a mobile application. The Love-Europe application helps them navigate, communicate, and integrate during their travels and stays. With the Love-Europe application, we offer a warm welcome to refugees in our countries and cities. For newcomers to Europe, the first priority is to understand the country and connect with people in Europe. Learning a new language is often a priority after arriving in one of the European countries. Getting to know the people and the culture is the key to integration” (Love-Europe, 2019).

The organization, whose main purpose is to help refugees migrating to European countries with issues such as language, housing, transportation, health, and weather through their smartphones, offers the application downloadable from iPhones produced by Apple. Currently, it provides the necessary locative data for refugees in Germany, the Netherlands, Switzerland, France, Greece, the UK, Austria, Portugal, Cyprus, Bosnia and Herzegovina, and Malta. The application, which provides general information about countries, is unique in detailing the rules to follow in each country (e.g., not littering, not smoking in public places, not bribing, not making noise in public places, avoiding physical contact, not walking in groups, and not responding angrily to racist incidents), what the locals like and dislike, as well as the general characteristics and cuisines of the local population. The application, which also has a website and a Facebook page, helps refugees find the most needed information.

Even when offline, the application continues to function for navigation and events. All data locations and activities are stored on the phone. In the absence of an internet connection, locations can be reached using the compass function. Due to its multilingual nature, the application can also be used as a communication tool.^1^ The conversation guide provides many basic sentence structures that can be used in another language. The application also provides information about local activities such as sports, cultural events, meetings, and celebrations, thereby accelerating the socialization process.

The data locations and map-based locations shown in the Love-Europe application allow refugees to easily move from their current location to their desired destination. Refugees using GPS can be guided through the applications and determine their next destination. When refugees reach certain areas such as big cities, they use the internet and GPS to go to places like refugee camps or bus stops. In interviews, refugees have emphasized the importance of using GPS applications such as Google Maps, describing the applications as “the best friend, the best thing, and the most necessary” for them (Zijlstra and Van Liempt, 2017).

### 4.2 The Refugee Buddy

**Figure 2:**
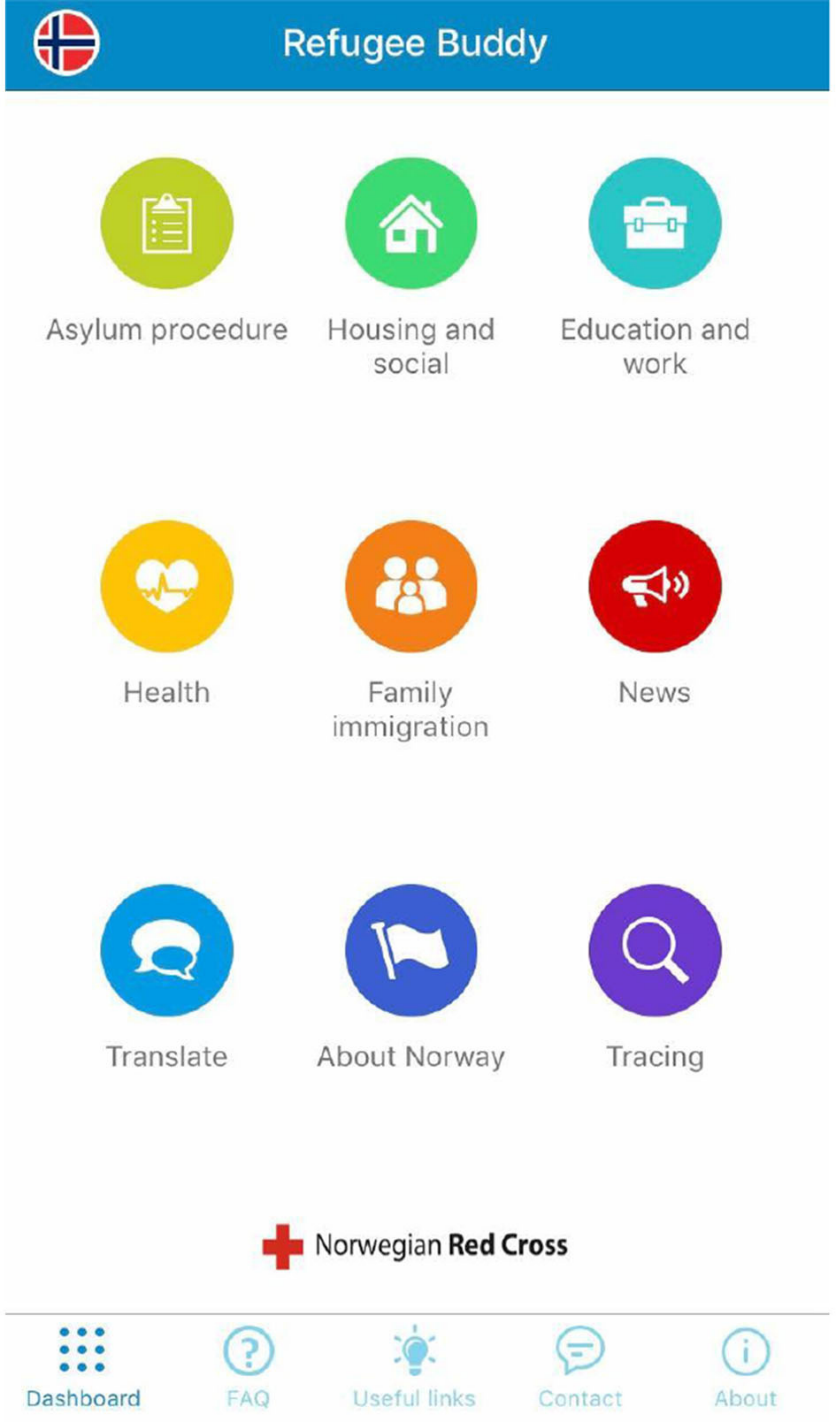
Refugee Buddy.

The Refugee Buddy application, supported by the Red Cross (Figure 2), can be downloaded via Apple iPhone. It is designed to assist refugees migrating to Canada, Cyprus, and Norway. After the refugee selects the country (Canada, Cyprus, or Norway), the application provides information about that country. The application is available in English, Arabic, French, and Tigrinya.^2^ On the first page of the screen, there are a total of nine separate applications related to asylum procedures, housing, education, employment, health, family reunification, news, translation, information about the country, and a search engine for the three countries.

For instance, when Norway is selected as the destination country, the asylum procedures page provides information on how to apply for asylum, where to apply, and the duration of the asylum-seeking process, followed by links to relevant websites. Similarly, on the housing, employment, health, family reunification, news, translation, and social support screens, the procedures to follow in Norway are detailed, with links to relevant web pages. Such applications are crucial for socialization in urban environments (Sutko Daniel and de Souza e Silva, 2011). Location-Based Social Networks (LBSN) increase socialization and coordination among people of similar socio-cultural backgrounds who prefer similar places, providing motivation among users (Şahoğlu-Tokgöz, 2018, p. 271). Thus, refugees or migrants can find individuals like themselves in the places they migrate to, making the integration process easier and establishing a connection with the new place more smoothly.

The application, which provides detailed information about countries, identifies the areas most needed by a refugee. Uncertainty and lack of sufficient information about the destination country can increase stress for a refugee. The refugee journey is described as a traumatic process reflecting their social world, mental and physical health (Mazzetti, 2008). The socialization and organization provided by locative media give refugees a sense of security (Schaub, 2012), reduce stress through the information obtained from applications, facilitate crisis management, and make it easier to reach a place through social connections (Alencar et al., 2018, p. 2). A study involving 16 refugees focused on their primary needs and the stress factors influencing them during the migration process, leading them to use mobile phones. According to the uses and gratifications theory, the study concluded that refugees use mobile phones for four primary purposes: as a friend, to communicate with associations, as a lifeline, and for entertainment (Alencar et al., 2018, p. 2–3). The influence of locative media reinforces the provision of these four needs, which serve security and socialization.

### 4.3 REFAID

**Figure 3:**
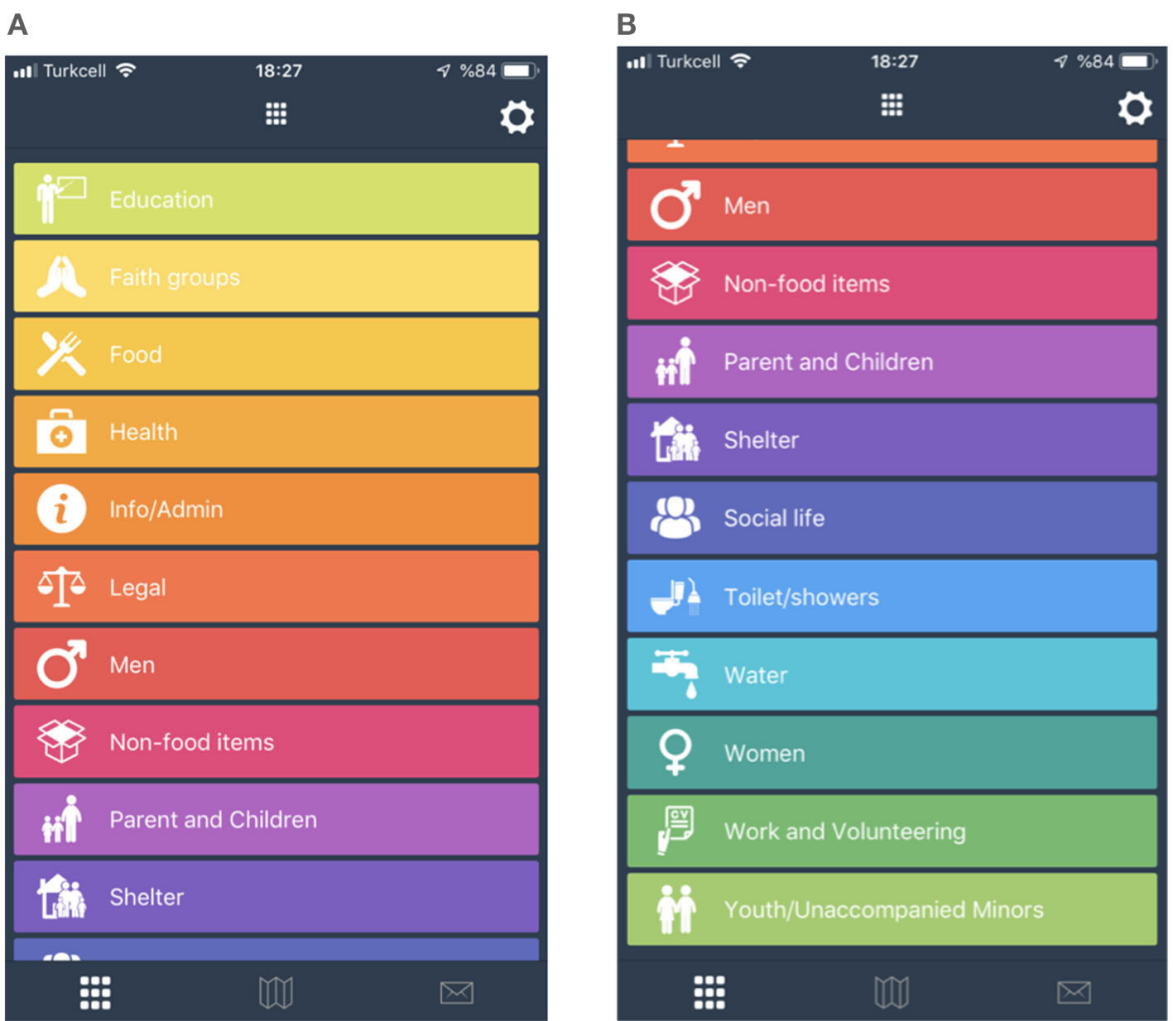
REFAID.

REFAID (Figures 3A, B) is designed to provide support, services, and information to refugees subjected to forced migration in English, Arabic, and Farsi. In addition to its mobile application, it also has a website, Twitter, LinkedIn, and Facebook accounts. The application primarily shows the nearest schools, food distribution centers, health centers, and locations of associations that provide solidarity with asylum seekers and immigrants as location data on a map. For refugees, the feeling of marginalization can be widespread in many host countries. Some associations established in the host country aim to reduce this feeling, providing counseling services upon request (IOM, 2008, p. 33). The purpose of the application is described on the REFAID (2019) website as follows:

“LifeSpots navigation is for migrants and refugees. The application shows aid locations on the map. All aid points shown in the application belong to reliable aid organizations. The application is currently available in Europe, the United States, and Türkiye. The application only shows what is available within 100 miles (150 km) of your location. There are more than 5,000 reliable service providers. Some services are offered in multiple languages, including Arabic and Farsi, in addition to English. More language options will be added soon. Don't worry - we won't give this information to anyone, and we won't tell anyone where you are. We just want to show you the nearest aid location on the map.”

Showing the locations of associations that help refugees and immigrants on the map can lead to two types of outcomes. First, it enables refugees to come together and socialize among themselves, which can hinder their integration with the local population. However, coming together and organizing through associations is valuable for feeling secure, protecting each other, and fostering solidarity, sharing, hope, and resistance. Refugees traveling by land and sea to an unknown country may face various dangers along the route, such as weather conditions, thirst, and hunger.

Applications help minimize the stress experienced by refugees. Location-based maps, showing the nearest shelters, food places, hospitals, transportation, and asylum locations, help reduce their stress. From this perspective, location-based data and maps that show where they are and where they need to go are extremely important for refugees. Knowing where they are and where to go through the application helps refugees feel secure (Alencar et al., 2018, p. 12). According to Sutko Daniel and de Souza e Silva (2011), spatial belonging and familiarity with the space make a person feel part of the place. According to research by Alencar et al. (2018, p. 11), refugees trust and act more on the information obtained from online refugee platforms. The REFAID application has a total of sixteen different topics of interest to refugees, each represented by a visual icon. The applications are well-designed visually, and with relevant website links, they ensure refugees are informed and their information needs are met in all areas. When examining the REFAID application below, different language options are evident. The initial impression created by the application is that it meets the needs of refugees. When refugees use the application, they access the desired information via links. The application pages are simple, clear, concise, and provide accurate navigation. It is crucial for a refugee to quickly find the information they need while browsing the application. The main topics are displayed in bold letters. Subheadings are relevant and easy to understand.

### 4.4 Find Hello

**Figure 4:**
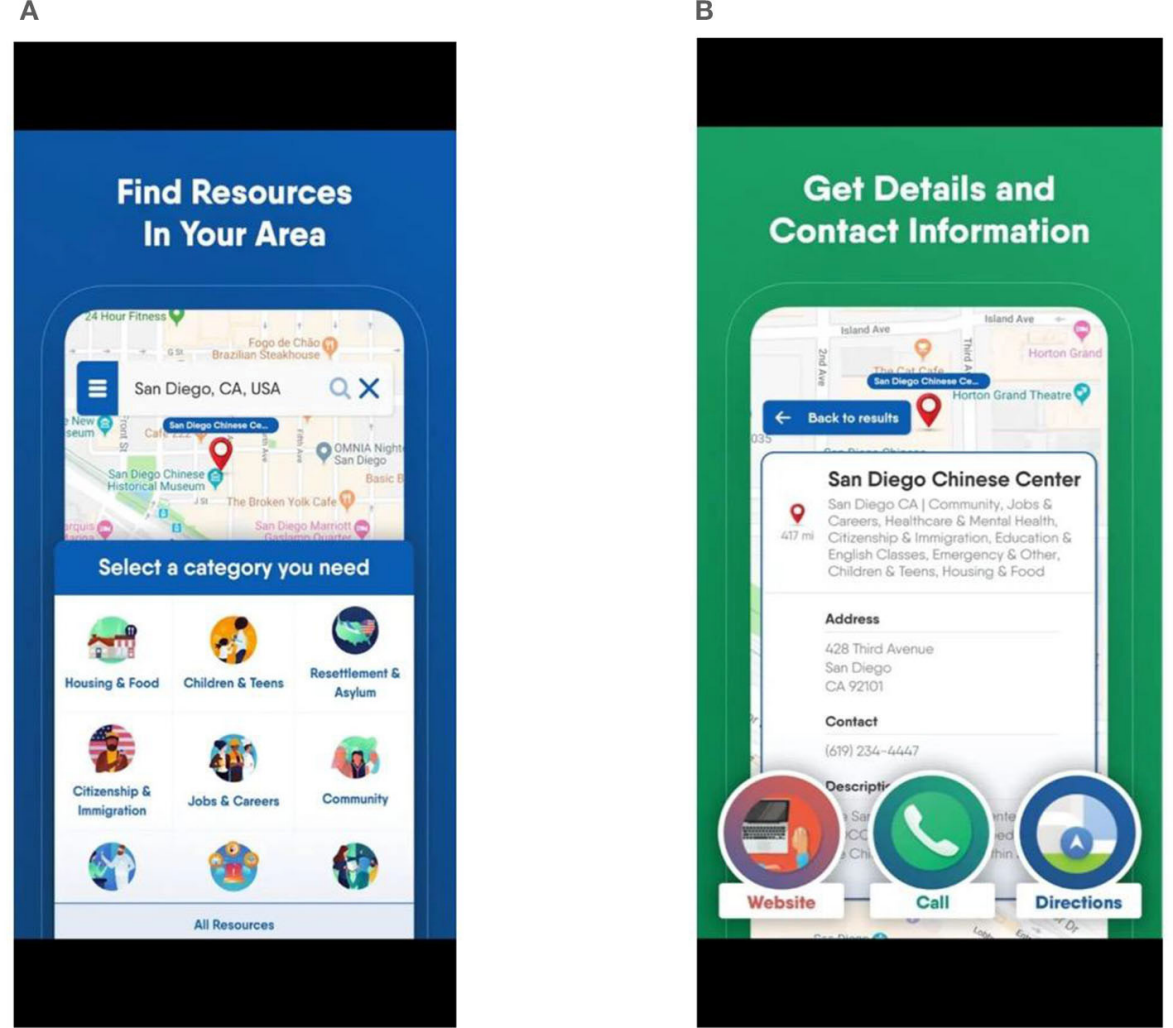
Find Hello.

The Find Hello application (Figures 4A, B) facilitates the easy discovery of local services for refugees, immigrants, and asylum seekers in the United States. The application, available in English, Arabic, and Spanish, is designed to be free and accessible to everyone without requiring registration. The homepage states that the application was created in partnership with the United Nations, addressing refugees, immigrants, and asylum seekers with the message: “Hello, welcome to America. You will find everything you need in this application. Whether you come from Mexico or Syria, Find Hello will assist you in establishing your new life in America.”

The application guides users in various areas such as job search, health, immigrant and refugee rights, education, scholarships, English classes, housing, food distribution locations, refugee associations, citizenship information, child and youth services, and the nearest emergency services. Providing all contact information instills a sense of security among participants. The ability to access phone, fax, or email information for contacting in case of any issues strengthens the sense of security and the connection established by the refugee.

### 4.5 I Stand With Refugees

**Figure 5:**
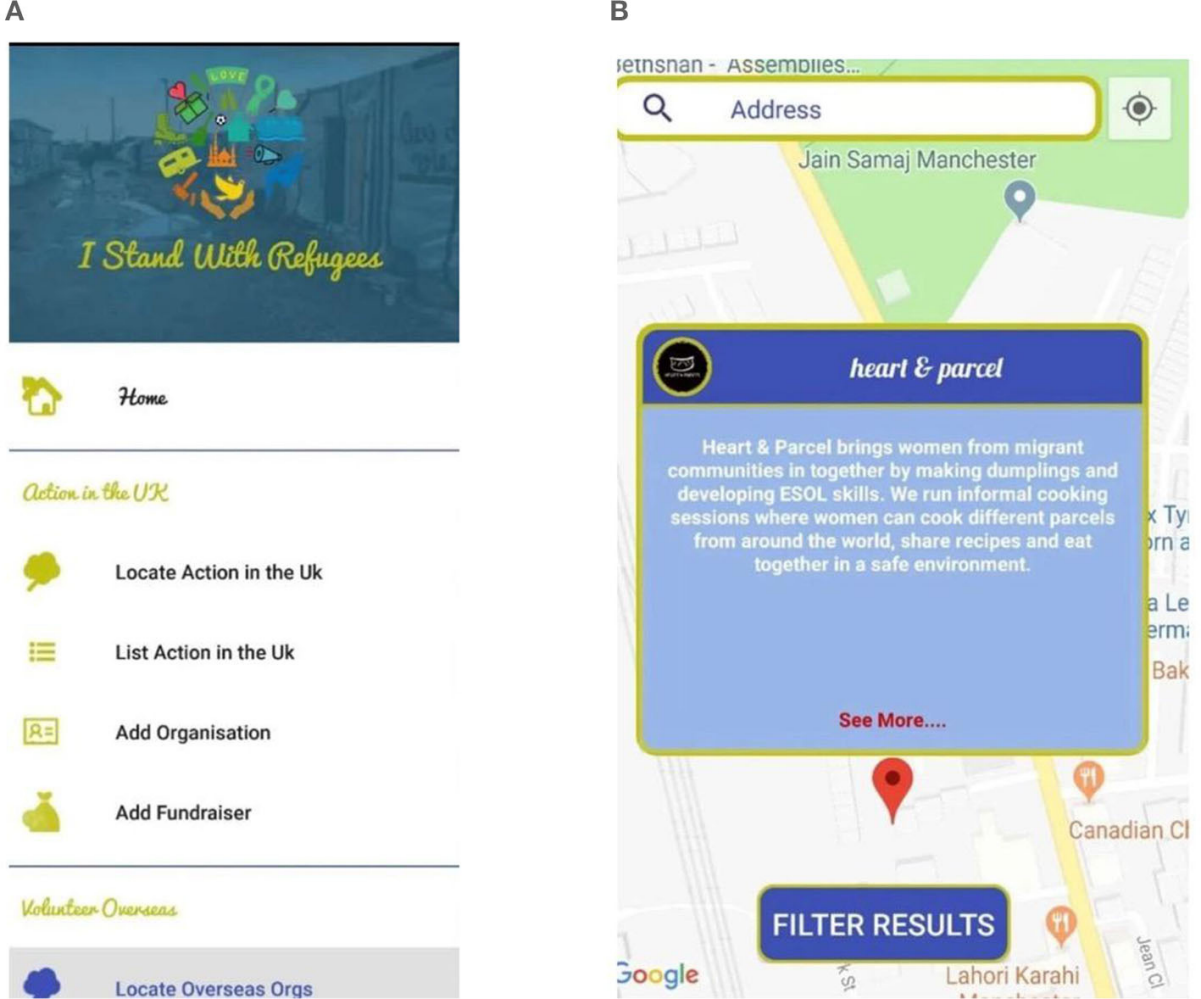
I Stand with Refugees.

I Stand With Refugees (Figures 5A, B) is an application designed for associations, aid organizations, and groups working with refugees to access accurate and instant information via smartphones. Used in the UK and many other countries, the application offers translation services, legal rights information, job opportunities, family reunification, language education, first aid, and fundraising for refugees fleeing persecution, poverty, and terrorism. The application's most notable feature is its location-based service, which shows the nearest services on a map by entering the current location's postal code.

Any refugee can learn about the necessary documents and legal processes for asylum or citizenship in the countries listed in the application before arriving there. This capability enables refugees to connect with other refugees, facilitating easy organization and experience sharing.

### 4.6 Integreat

**Figure 6:**
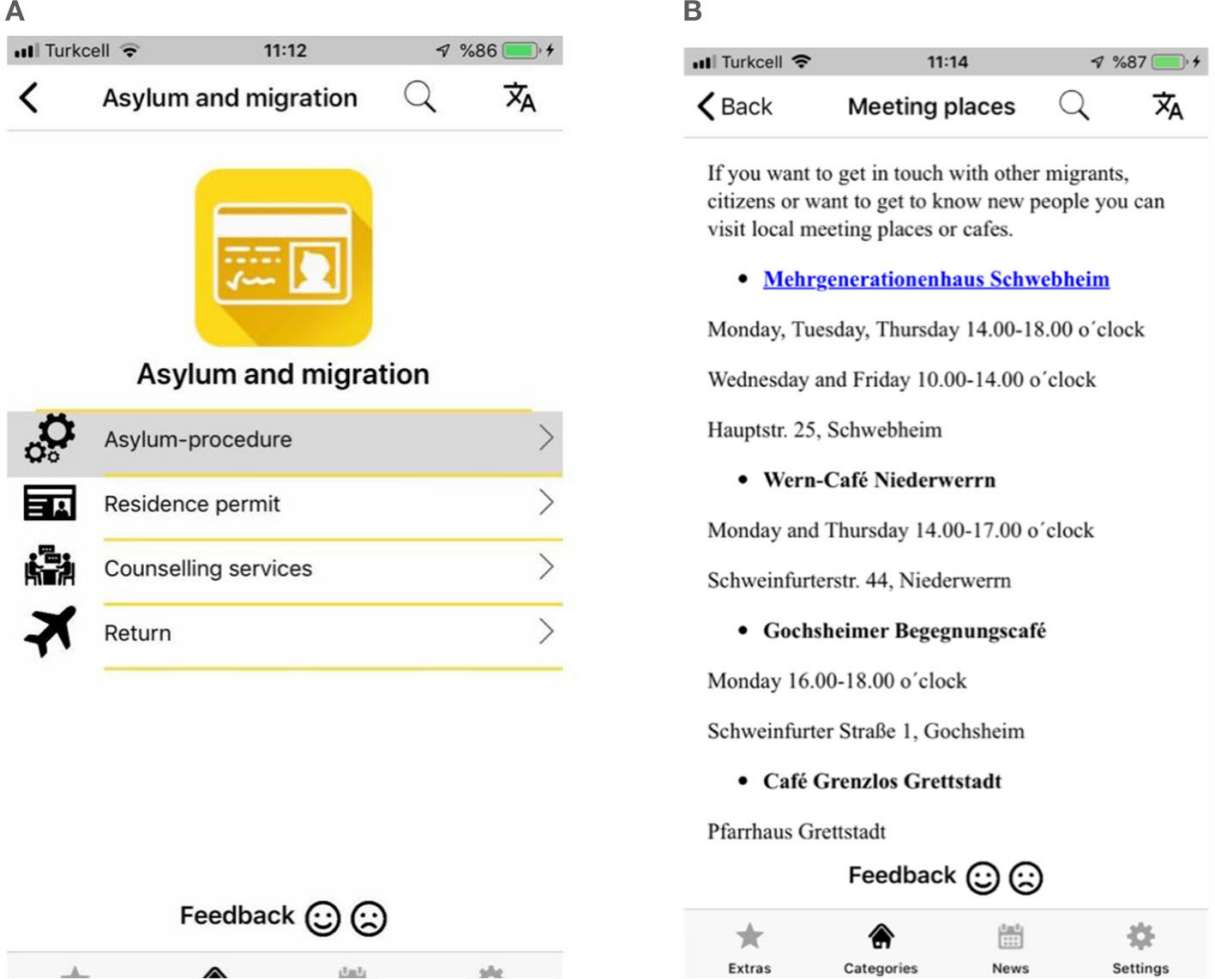
Integreat.

In 2015, after seeing a flyer from a refugee association (Tür an Tür), Daniel Kehne developed the Integreat application (Figures 6A, B) to facilitate the integration of newcomers and refugees in Germany. Social workers, scattered throughout cities like Augsburg, faced difficulties in providing effective and essential information on transportation, health, asylum rights, employment, education, and legal rights to refugees who did not speak German. This need for effective communication led to the creation of the Integreat application.

Integreat collaborates with local social service experts, NGOs, and other stakeholders in over 80 cities across Germany to prepare specialized content for each area. Working in cooperation with students and volunteers throughout Germany, the application is designed to provide important information in five languages (German, English, Arabic, Farsi, and French) to asylum seekers arriving in unfamiliar environments, particularly in Augsburg.

The homepage of the Integreat application features main headings for a total of 53 regions, covering topics such as welcome, asylum and migration, language, education, work and university, family, health, daily life, and emergency procedures. The initial greeting for newcomers to the page warmly welcomes them. Clicking on any region's link provides access to information specific to that area. The page includes municipalities, maps of the region, and key departments (health, immigrant and refugee offices, employment agency, youth aid office, social welfare office, registration center, immigration office).

Examining the welcome page for Schweinfurt, the following message appears, accompanied by a photo of District Manager Florian Töpper:

“Welcome to the Schweinfurt region! It is not easy to navigate your way in a new culture, country, or city. With this application, we aim to assist you during your first months in Schweinfurt. Integreat, with the support of volunteer workers, aims to provide you with an overview of important information and contact numbers. This application is a tool for asylum rights, learning German, education, employment, and making contacts. We hope that Integreat helps answer some of your questions and find solutions in this region. We are happy to welcome you to the Schweinfurt region. Best wishes. (31.01.2019)”

The welcome application page also provides information about citizen groups. These groups, entirely composed of volunteers, help refugees find accommodation, participate in recreational and sports activities, learn German language skills, translate German texts, find cheap shopping places, locate bus stops, assist children with homework, and answer questions related to bank accounts. Similarly, the welcome page provides links to the websites of all municipalities in the region and shows their locations. The health department section of the welcome page includes information on promoting health and preventing addiction, pregnancy and childbirth, family planning, contraception and sexuality, HIV/AIDS counseling, school registration exams, medical exams, and vaccination counseling. The Federal Office for Migration and Refugees (BAMF) is responsible for implementing asylum procedures, making decisions on asylum applications, supporting projects and programs for social integration (e.g., integration courses), prevention (e.g., a counseling center against radicalization), and funding programs that return people to their previous countries.

The Employment Agency section on the welcome page provides support for those whose asylum procedures are still ongoing or who believe they have been wronged. The Federal Employment Agency assists with the transition from school to work (career guidance), job placement, vocational training counseling, and employer advice. Additionally, the Employment Agency is responsible for refugees when they have a residence permit and are not receiving services from the Job Center. The Job Center, linked on the same page, serves as the contact point for the unemployed or those in need of assistance. It provides financial support, helps with reintegration into the job market, finds language courses, assists with career choices and recommendations, and helps identify and recognize qualifications. The page provides a step-by-step list of what those granted asylum should do. Those who receive a positive result from BAMF must follow three steps: personal interview, service department, and employment agency. The Youth Welfare Office on the welcome page supports parents and guardians in raising, caring for, and educating children and young people. It offers protective and family-friendly services to create positive living conditions for families.

The applications mentioned above, which contribute to the formation of hybrid spaces, facilitate communication and coordination among refugees, local people, and local governments. Applications developed jointly by NGOs, volunteers, and governments not only provide refugees with information about the places they will go but also make socialization easier. Socialization or integration is a two-way process in which both the refugee and the host society have responsibilities toward each other (IOM, 2008, p. 21). Maintaining social solidarity can be considered one of the primary goals of integration. In our time, the ability and capacity of states to host, integrate, or deport migrants are perceived as indicators of their ability to protect and develop their citizens and territories (Anderson et al., 2011, p. 543). Preventing the marginalization and exclusion of refugees is essential not only for the welfare of migrants but also for the functioning and social stability of the host country. Well-integrated refugees are more likely to fulfill their potential and make positive contributions to the economic, social, and cultural life of the host country (IOM, 2008, p. 17).

### 4.7 OKA

**Figure 7:**
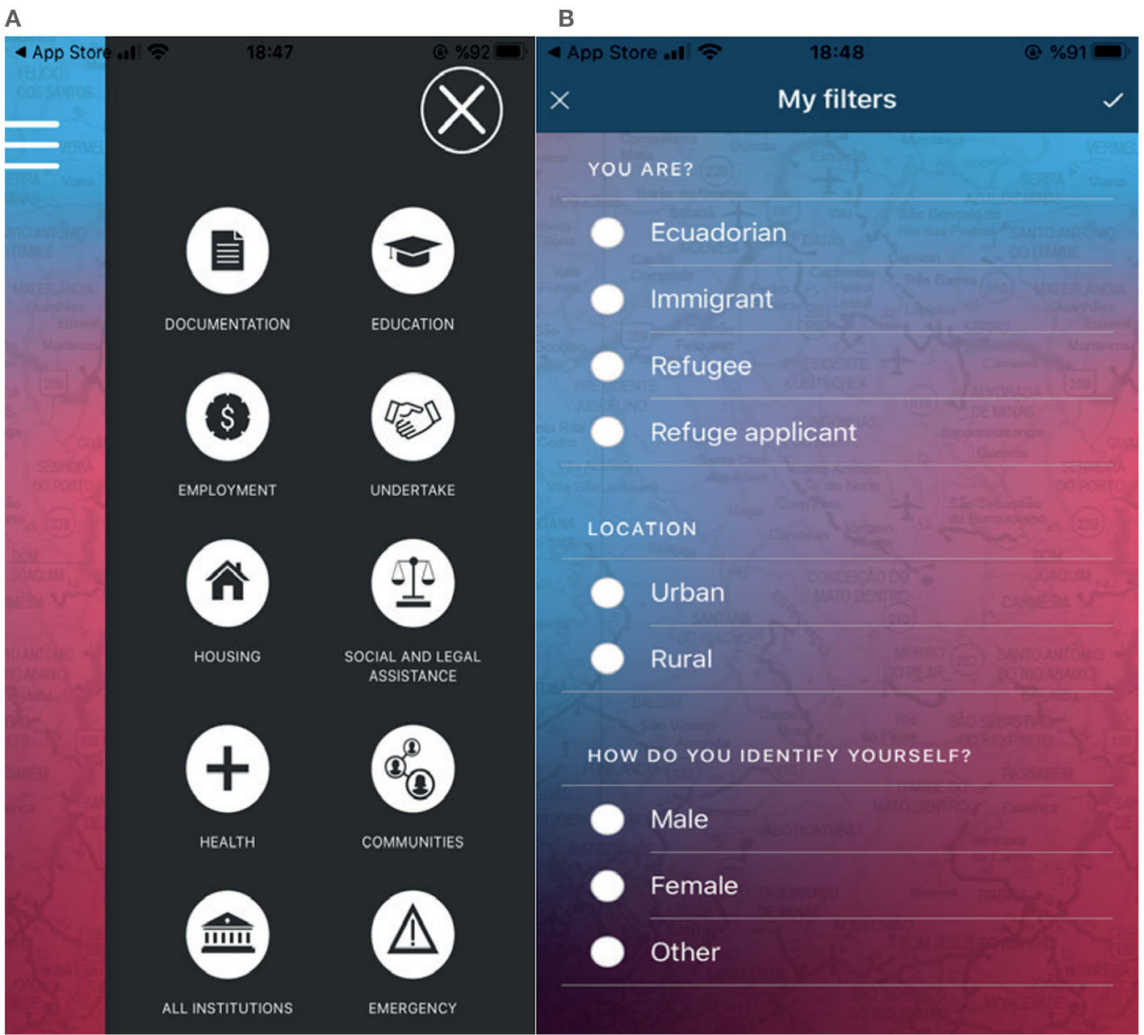
OKA.

OKA (Figures 7A, B) is an Android and iOS mobile application for immigrants, refugees, asylum seekers, and internally displaced persons in Brazil. It is available in Portuguese, Spanish, French, and English. Developed in collaboration with refugee and immigrant communities, OKA is designed to meet the specific needs of refugees. The application can be used both online and offline and provides detailed information about services offered by municipal and public institutions across Brazil, particularly in São Paulo, Rio de Janeiro, and Boa Vista. An updated version of the application was launched on International Human Rights Day at the Migration Museum in São Paulo. A renowned musician, Nando Reis, shared a video about OKA that reached over 32,000 people (Igarapé Institute, 2020).

Following the economic and political crisis in Venezuela in 2013, thousands of Venezuelans migrated to neighboring countries, primarily Brazil. Between 2015 and 2017, 44,000 Venezuelans entered Brazil through the northern state of Roraima, reaching its capital, Boa Vista. This influx exacerbated the existing unemployment rate in Brazil (UNHCR, 2019). Since 2017, ~100,000 new refugees, primarily from Venezuela, have arrived in Brazil. The biggest challenge for refugees is accessing basic services. To inform Venezuelan refugees about their rights, provide information about the labor market, find suitable positions for job seekers, and educate them about the health and education systems, discussions began on what needed to be done. To overcome this challenge, the Igarapé Institute launched OKA, a free mobile application. OKA integrates geographically positioned critical information for refugees, asylum seekers, and internally displaced and vulnerable communities on housing, food sources, education, transportation, legal assistance, employment, and healthcare services (Igarapé Institute, 2020).

### 4.8 Merhaba Umut

**Figure 8:**
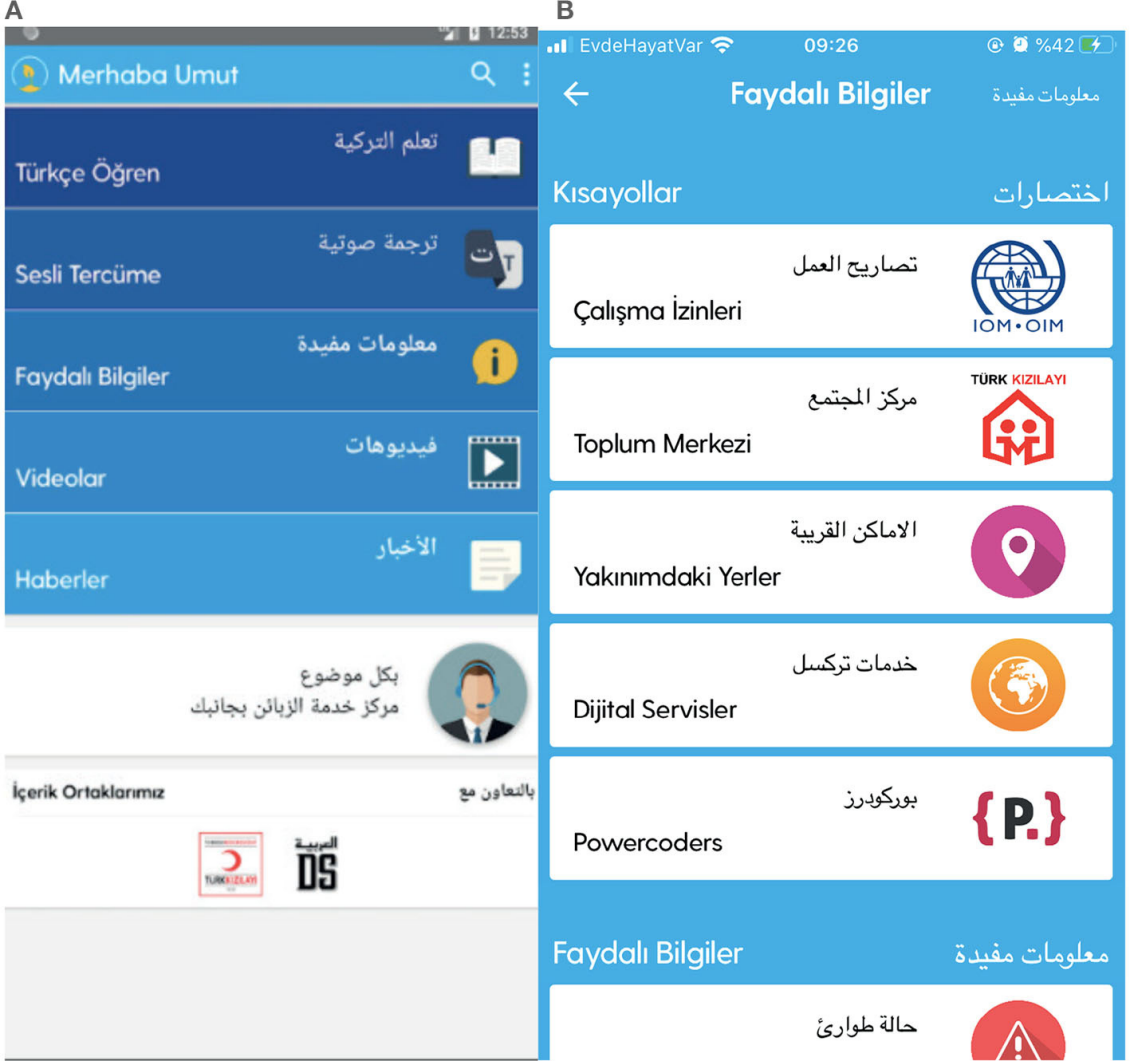
Merhaba Umut.

The “Merhaba Umut” (Hello Hope) application (Figures 8A, B), developed by Turkcell Bilişim Servisleri A.S., is a free mobile app available for Android and iOS, designed to facilitate the lives of Syrians in Türkiye and help them adapt to Turkish culture. The app was created in response to the mass migration of Syrians to Türkiye following the escalation of anti-regime protests into conflicts in Syria in March 2011. Official records indicate that there are 3,605,152 Syrians under temporary protection in Türkiye (Göç Idaresi Genel Müdürlügü, 2020).

Temporary protection is a provision granted by the State to individuals fleeing conflict or violence in significant numbers, without undergoing individual status determination procedures. According to the 2004 UNHCR Executive Committee's Decision No. 100, for a situation to be considered mass refuge, there must be significant and continuous movement of people toward another country's border, overwhelming the host country's capacity to follow existing individual asylum procedures. In such cases, temporary protection is provided. It is a practical solution for mass influxes, ensuring non-refoulement obligations are met without delay (Göç Idaresi Genel Müdürlügü, 2020).

Türkiye's temporary protection for Syrians includes three main elements: open border policy, non-refoulement, and meeting the essential and urgent needs of those arriving. The protection provided is in accordance with Article 91 of the Law on Foreigners and International Protection No. 6458, dated 04/04/2013, and the Temporary Protection Regulation No. 2014/6883, dated 13/10/2014 (Göç Idaresi Genel Müdürlügü, 2020).

The “Merhaba Umut” app was developed to address the issues faced by Syrians in Türkiye and to expedite their integration process. Its primary purpose is to meet the communication needs of Arabic speakers in Türkiye. Users can learn the most commonly used words and phrases in Turkish, both written and spoken. Additionally, the app provides location-based services to help users find the nearest registration offices, health services, and other essential facilities.

In the Useful Information section, the app offers critical details on various aspects of daily life in Türkiye, including official procedures, health, education, travel, transportation, accommodation, and support services. It also provides directions to the nearest official institutions, hospitals, pharmacies, banks, mosques, police stations, and bus stops, showing their names, addresses, and distances on a map (Merhaba Umut, 2020).

The app's main page includes sections for learning Turkish, voice translation, useful information, and news. The Learn Turkish section is divided into two parts: one for children and one for everyone. For children, the sections include alphabet, greetings, food, numbers, colors, animals, dates, and time. For instance, the greetings section for children contains 40 phrases in Turkish and Arabic, such as “Which school do you go to?”, “What grade are you in?”, “Do you have any siblings?”, “Do you have brothers or sisters?”, and “How many siblings do you have?” The food section includes categories for vegetables, fruits, and general foods.

The section for everyone includes categories such as alphabet, emergency, basics, greetings, health, transportation, food, professions, accommodation, dates, seasons and months, numbers, and currency. The emergency section contains 45 phrases, including “Help, I am in danger,” “Please help me,” “Rescue me,” “Be careful,” “Call the police,” “I am lost,” “Ambulance,” “Fire,” and “Call the fire department.” The basics section covers words, family, greetings, congratulations, colors, animals, weather, clothing, and household items, providing daily information in Turkish and Arabic. The section on work permits in the Useful Information section includes details about temporary protection, international protection, and applications for domestic and international work permits. Information is available in both Turkish and Arabic, and is also presented in animated video format. The community centers section within Useful Information details courses provided to immigrants and refugees under the adaptation and livelihood improvement program, along with address and contact information for community centers across Türkiye. The Nearby Places section in Useful Information lists locations for schools, universities, post offices, banks, mosques, apartments, hospitals, pharmacies, doctors, dentists, bus stops, metro stations, train stations, and museums.

### 4.9 I'MAPPY

**Figure 9:**
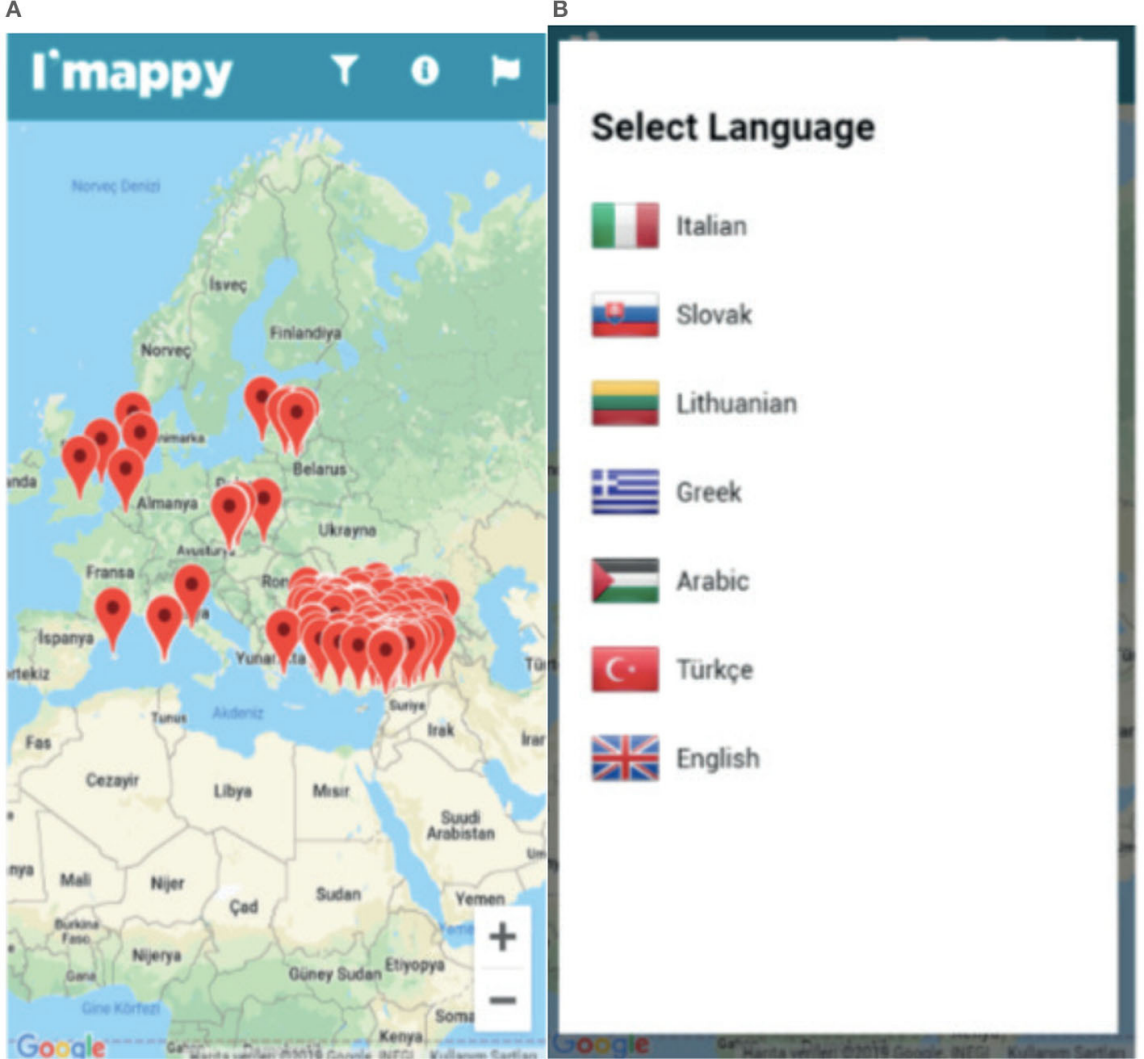
I'Mappy.

The Integration Map and Enhanced Network for Refugees (I'MAPPY) (Figures 9A, B), developed by the Research Center on Asylum and Migration (IGAM), is an Erasmus+ project funded by the National Agency for 24 months, starting on February 1, 2017, and ending on January 31, 2019. The project, led by IGAM, included six partners from five countries: Tera Ankara (TR), VsI Pasaulio Pilieciu Akademija (LT), A.D.E.L (SK), IASIS (GR), and TDM 2000 (IT). I'MAPPY is available in Italian, Lithuanian, Greek, Arabic, Turkish, and English.

The primary idea behind the project was to create an integration map for young refugees without social and basic information access in their host country, especially those without parents or families. I'MAPPY provides language courses containing social and basic information for young refugees. Additionally, the application identifies and collaborates with NGOs that support minority groups and offers information on placing migrant workers in jobs and engaging employers (I'MAPPY, 2020).

### 4.10 Migrant Information Center

**Figure 10:**
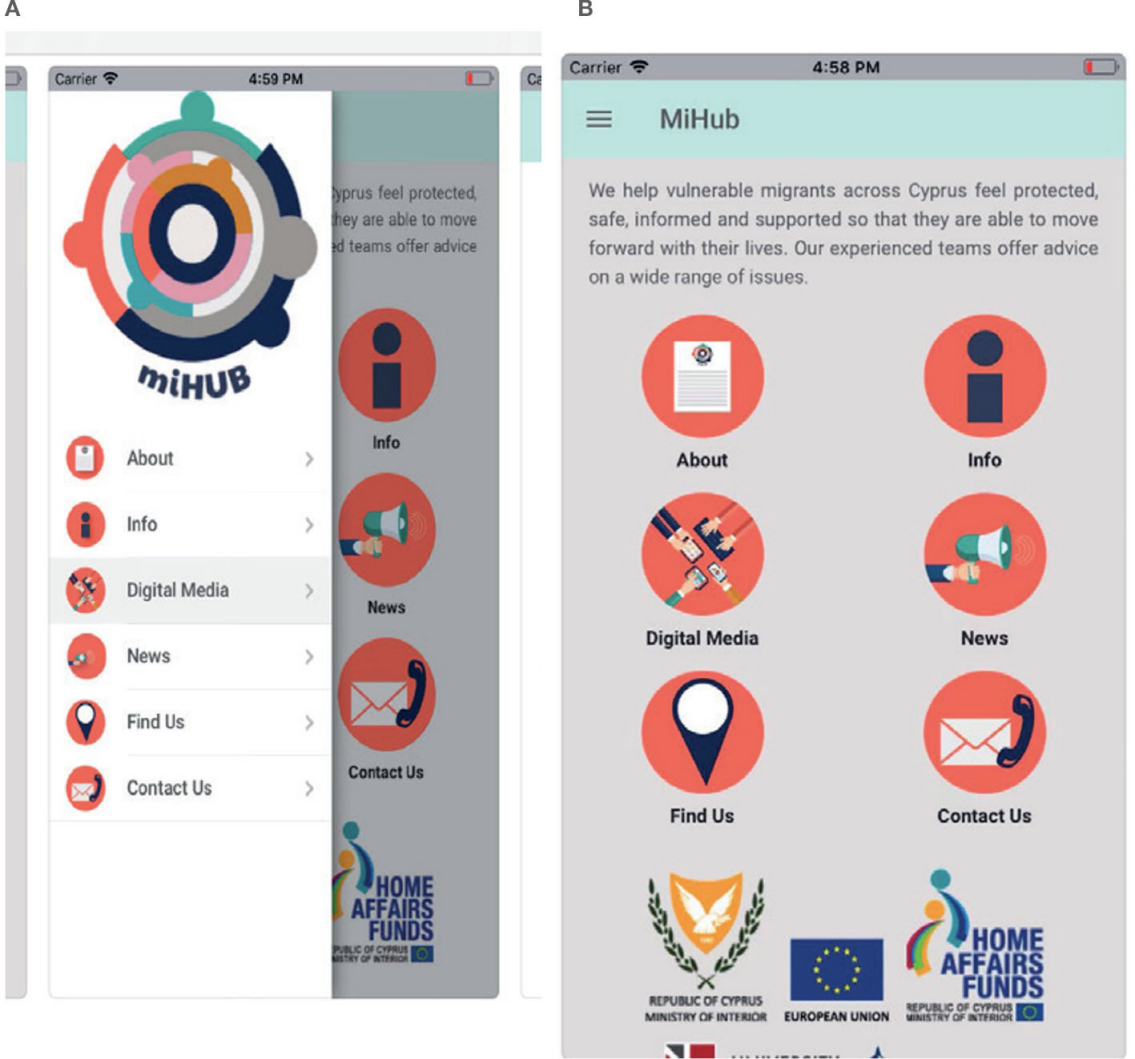
MiHUP.

The Migrant Information Center (MiHUB) application (Figures 10A, B) is designed to assist migrants, asylum seekers, refugees, beneficiaries of international protection, and third-country nationals in Cyprus. It provides information on housing, education, health, rights and responsibilities, employment, learning English and Greek, travel, social benefits, and counseling centers. The application was developed in collaboration with CARDET (Center for the Advancement of Research & Development in Educational Technology) and Cyprus University of Technology. It values protection, diversity, equality, cooperation, innovation, and excellence.

In the housing section, the application discusses the benefits of international protection status. According to the information provided in the app and on the website, asylum seekers have the legal right to reside freely in the Republic of Cyprus based on the Refugee Law (6 (I) 2000). The application mentions that there is only one Reception Center for asylum seekers in Cyprus, located in Kofinou, with a capacity of about 400 people. Asylum seekers can stay at the center temporarily for up to 6 months until they find more suitable accommodation. The Asylum Service, responsible for the operation and supervision of the center, prioritizes vulnerable groups such as families with young children, single women, or women with children. Services include free meals, financial support, free transportation services, and schooling for children of families residing at the center (MiHUB, 2019).

In the education section, the application highlights the importance of education and language in the integration process. The health section discusses the legal framework and health services, stating that under the Law on Refugees (2000), asylum seekers without means of support have the right to free care. Refugees have the same rights as Cypriots, so the same criteria apply for free hospital services. MiHUB also provides detailed information on topics such as who qualifies as an asylum seeker, their legal rights, the detention process, and locations of detention centers (MiHUB, 2019).

The application features an important announcements section that includes information on the extension of COVID-19 measures. It mentions that the Republic of Cyprus decided to extend the measures to combat the COVID-19 pandemic until April 30, 2020. Asylum seekers are advised to inform the authorities by calling the number 8998 or filling out Form B before traveling (MiHUB, 2019).

## 5 Conclusion

The advantages of technology in the digital age, such as locative media, assist refugees in navigating their arduous and perilous migration journeys, communicating with other refugees, coordinating their actions, determining their routes, finding direction, adapting to the country, and ensuring their safety in the city. Recognizing the situation, many countries affected by migration have developed applications that facilitate the lives of thousands of refugees entering their territories and ensure spatial awareness. Initially developed by volunteers, these applications have since expanded to include non-governmental organizations, the United Nations, and private companies. When these applications, which also enhance solidarity and integration, are examined, it is understood that they contain all the information any refugee might need. They provide basic information such as health, employment, asylum rights, renting a house, school information, transportation information, asylum points, translation, and residence permits, while also including location data with GPS maps, thereby accelerating the refugee's adaptation process.

In the study, a total of ten location-based applications for refugees were examined. The primary reason for selecting these ten applications is that they are used in the countries receiving the highest number of immigrants and help to address the issues that refugees might encounter in these countries. Additionally, the presence of location-based media in these applications, which provides essential information for refugees such as finding jobs, housing, healthcare, education, language, behavioral norms, maps, psychological support, institutional information, transportation, and basic needs, answers our research questions. All this essential information is capable of positively influencing the adaptation process of refugees to a new country. Moreover, such applications contribute to overcoming the challenges faced by refugees and aid them in starting a new life. The effective use of technology during the adaptation process is an important tool in supporting the social integration of refugees.

The study is significant for several reasons. Firstly, it emphasizes the need for digital examination of migration research. With the advancement of digital technologies, research methods have acquired a digital dimension. A person planning to migrate now gathers information about the destination country through social media and applications before even arriving. Thus, the migration stories of refugees are being rewritten with digital data, not just limited to official records or news. On the other hand, digital literacy, which refers to the ability to effectively use digital devices, the internet, and digital tools, plays a critical role in the integration process of refugees. Therefore, increasing support programs and training in these areas for refugees will help them adapt more quickly to a new life.

Secondly, it emphasizes the importance of smartphones and applications for refugees in today's world. According to research, such applications accelerate the process of solidarity, adaptation, socialization, and integration among refugees. The study's limitation lies in examining only the content of ten applications. Due to the limited sample size, the findings are not claimed to represent all refugee applications. In future studies, the generalizability of the findings could be increased by using a larger sample. In future research, more detailed comparisons could be made of the applications analyzed. For example, by making more comprehensive comparisons between the effects of applications used in different countries, it could be determined which features are more effective. Additionally, the impact of the design features of the applications on refugee adaptation could be examined in more depth.

On the other hand, the lack of sufficient theoretical research on digitalization, location-based applications, migration, and refugees emerges as a significant deficiency in the field of study.

Digital communication technologies, which eliminate the boundaries between time and physical space and create hybrid spaces, offer significant insights for interdisciplinary studies. The internet and social networks also lead to the formation of collective intelligence. Therefore, future research on this topic needs more field studies and the examination of cyberspaces. This study suggests investigating what is lacking in applications developed for refugees, whether they affect solidarity and socialization, why volunteers develop such applications, and what additional information should be provided specifically for female refugees.

## Data Availability

The original contributions presented in the study are included in the article/supplementary material, further inquiries can be directed to the corresponding author.

## Footnotes

^1^Language is a precursor to all other aspects of integration. The ability to speak the language of the host country is one of the most critical aspects of integration. Language and communication help establish bonds that facilitate integration between refugees and members of the host society, forming the basis for participation in the social, cultural, and political life of the host country (IOM, 2008, p. 34).

^2^A significant issue for refugees is the inability to speak the language of the country they migrate to. Applications developed to address this issue instantly eliminate language barriers through volunteer workers. One such application, Tarjimly, can be downloaded on iOS or Android, requiring registration. The application has registered 21,637 users, including 18,359 translators, 11,963 aid workers, and 9,674 refugees. Supported in 103 languages, the most commonly used languages are English, French, Arabic, Farsi, and Urdu. According to official data, the application usage by topic is distributed as follows: 27.9% health, 18.9% camp needs, 18.9% legal regulations, 13% education, 10% asylum application, 4% employment, 1% food, and 0.8% psychological issues. Aid workers can also use Tarjimly and register their organizations. When a refugee needs a translator, they can search for and connect with an available volunteer by selecting the required language in Tarjimly. Depending on the language pair, this process can take between 30 s and 5 min. Refugees can connect with the volunteer worker via live video and can also leave documents, text, or voice notes for translation (Tarjimly, 2019).
